# Vitamin D deficiency among newly diagnosed tuberculosis patients and their household contacts: a comparative cross-sectional study

**DOI:** 10.1186/s13690-017-0195-7

**Published:** 2017-06-19

**Authors:** Meseret Workineh, Biniam Mathewos, Beyene Moges, Adissu Gize, Sisay Getie, Olle Stendahl, Thomas Schon, Ebba Abate

**Affiliations:** 1 Department of Immunology & Molecular Biology, School of Biomedical and Laboratory Sciences, University of Gondar, Gondar, Ethiopia; 2 Department of Medical Microbiology, Linkoping University, Linköping, Sweden; 3 Department of Clinical Microbiology and Infectious Diseases, Kalmar county Hospital, Kalmar, Sweden; 4 St. Paul’s Millennium Medical College, Addis Ababa, Ethiopia; 5 Department of Medical Parasitology, School of Biomedical and Laboratory Sciences, University of Gondar, Gondar, Ethiopia

**Keywords:** Vitamin D deficiency, Tuberculosis, Ethiopia

## Abstract

**Background:**

Recent studies suggest that the incidence and severity of tuberculosis is associated with low levels of vitamin D. Even though individuals living in Ethiopia have a high exposure to sunlight which is a source of vitamin D, tuberculosis is still one of the major causes of morbidity and mortality in the country. Therefore, this study aimed to determine the prevalence and associated factors of vitamin D deficiency in newly diagnosed tuberculosis patients, household contacts and community controls in Gondar, Ethiopia.

**Methods:**

A comparative cross-sectional study design was conducted. Blood samples were collected from newly diagnosed smear positive pulmonary TB patients, their household contacts and community controls. Serum 25(OH)-vitamin D_3_ was determined by an Enzyme Linked Immunosorbent Assay. A serum level of 25(OH)-vitamin D_3_ below < 50 nmol/L was defined as vitamin D deficiency and <25 nmol/L as severe vitamin D deficiency.

**Results:**

A total of 126 newly diagnosed smear positive TB patients, 57 house hold contacts and 70 apparently community controls were included in the study. The mean ± SD age (years) of TB patients, house hold contacts and community controls was 29.8 ± 11.9, 24.3 ± 14.7 and 27.3 ± 7.6 respectively. Ninety out of 126 (71.4%) TB patients were underweight with a BMI of < 18.5 kg/m^2^. The mean 25(OH)-vitamin D_3_ level of TB patients (30.1 ± 19.3 nmol/L) was significantly lower than community controls (38.5 ± 20.9 nmol/L, *P* = 0.005 and household contacts (37.7 ± 12.8 nmol/L, *P* =0.031).). The prevalence of vitamin D deficiency was higher in TB patients (83.3%) than in community controls (67.1%, *P* = 0.009). The prevalence of vitamin D deficiency was also found higher in household contacts (80.7%). Severe vitamin D deficiency was observed in 53%(67/126), 30% (21/70), 19.3%(11/57) of TB patients, community controls and household contacts respectively. Low BMI (AOR = 2.13; 95%CI: 1.02, 3.28) and being positive for tuberculosis (AOR = 1.93; 95%CI: 1.06, 2.86) were significant predictors of severe vitamin D deficiency.

**Conclusion:**

High prevalence of vitamin D deficiency was found among newly diagnosed TB patients and in their household contacts. The present study warrants further studies to determine the role of vitamin D supplementation in the prevention and treatment of tuberculosis in Ethiopia.

## Background

Tuberculosis (TB) is an infectious disease caused by the bacilli belonging to *Mycobacterium tuberculosis* complex, usually *Mycobacterium tuberculosis (MTB)* [1]. TB remains a major global health problem. According to World health organization (WHO) global tuberculosis report, 9.6 million TB cases occurred and TB killed 1.5 million people (1.1 million HIV-negative and 0.4 million HIV-positive) worldwide in 2014 [2]. Ethiopia has ranked 7^th^ among the world’s 22 high burden countries with an estimated incidence rate of 207 per 100,000 population in 2014 [2].

The host susceptibility to TB infection depends on a complex interaction between host, bacterial as well as environmental factors, such as poverty, malnutrition, overcrowding, and exposure to other pathogens [3, 4]. In addition to environmental factors, genetic factors, such as polymorphisms in the interleukin-1 (IL-1) gene cluster and mannose-binding lectin have been shown to influence host susceptibility to TB [5]. Cell-mediated immunity is important for host protection against mycobacteria infection [6].

Vitamin D is synthesized in the skin during exposure to ultraviolet light and is also available in the diet, principally from fish [7]. Vitamin D has found to play an important role in the host immune defense against TB by improving phagocytic capacity of monocytes and macrophages [8, 9], increasing the production of antimicrobial peptides such as cathelicidin [10], and by immunomodulatory effects [11]. Vitamin D deficiency in mice resulted in the increased replication of *Mycobacterium bovis* [12]. Studies have also shown that vitamin D induces a superoxide burst and enhances phagolysosome fusion in MTB -infected macrophages [13–16]. Some studies have also shown that polymorphisms in the vitamin D receptor influence host susceptibility to TB [5].

Other groups have shown that vitamin D inhibits the generation of Th1 responses and the production of Interferon-γ by promoting the generation of regulatory T cells (Tregs) [12]. Thus paradoxical effects of vitamin D have been observed in immunity of tuberculosis: decreased vitamin D mediated Th1 immunity, but increased bactericidal activity [17]. Given that the Th1 response is protective, but also causes pathology, vitamin D may provide the ideal response by inducing increased bactericidal activity coupled with a decreased, but present, Th1 response.

Several studies have shown conflicting results on the level of vitamin D in TB patients and in community controls [18–30]. Serum vitamin D level varies considerably between populations and is influenced by many geographical and cultural factors [31, 32]. Although individuals living in Ethiopia have a higher chance of exposure to sunlight; which is the main source of vitamin D, tuberculosis is still one of the major causes of morbidity and mortality in the country. There is no previous data showing the prevalence of vitamin D deficiency and associated factors in newly diagnosed TB patients compared with apparently healthy community controls in the study area. Furthermore, no previous study has investigated the prevalence of vitamin D in household contacts of TB patients in Ethiopia. Thus, the aim of this study was to determine the prevalence and associated factors of vitamin D deficiency in newly diagnosed TB patients compared to household contacts and community controls in Gondar, North West Ethiopia.

## Methods

### Study design and area

An institutional based comparative cross sectional study was conducted from January 2013 to May 2013 at the University of Gondar Hospital (GUH) and Gondar Health Center (GHC), Northwest Ethiopia. Gondar University hospital is a tertiary level teaching and referral hospital with 450 beds for inpatients and rendering referral health services for over 5 million inhabitants in North-West Ethiopia. The hospital provides inpatient and outpatient services to the population in the surrounding area of Gondar town and the adjacent regions. The hospital has TB clinic where TB patients are getting their medication and further assessment during follow up period. Gondar health center is also located in Gondar town and it provides different health services to population of Gondar town and the surrounding area. The health center has a TB clinic where TB patients are getting treatment through the Directly Observed Treatment Short-Course (DOTS) programme.

### Study population

Consecutive newly diagnosed smear positive tuberculosis patients were recruited at the DOTS clinics of the two health facilities. Smear positive TB was defined as at least two sputum smears positive for acid fast bacilli (AFB) or one smear positive slide and x-ray results suggestive of TB [33]. Apparently healthy blood donors who visited the blood bank at the GUH during the study period, and had passed pre-donation clinical screening to rule out any chronic illnesses and previous TB history, were enrolled as community controls (CC). In addition, household contacts (HHC) livings together with the index TB cases were included. A household contact was defined as a person who lives together (>6 months) and spends more than 12 h per day with a TB patient. HIV screening was routinely performed in voluntary counseling and testing clinics (VCT) at GUH and GHC for all TB patients. HIV positive tuberculosis patients were excluded from the study in order to enable direct comparison with blood donors as community controls.

### Data collection

Socio-demographic data were collected using a structured and pretested questionnaire. Data on age, sex, level of education, occupational status, smoking, and alcohol intake were collected from all newly diagnosed TB patients and community controls. All household contacts had data on age and sex; but not regarding level of education, occupational status, alcohol use and tobacco use. Height and weight were measured for all TB patients, household contacts and community controls, and body mass index (BMI) was calculated using the formula: weight (kg)/height (m^2^). Blood samples were collected for 25(OH)-vitamin D_3_ measurement, and the serum was harvested immediately following centrifugation at 3000 rpm, and stored at −20 °C.

### Measurement of vitamin D

The serum vitamin D was measured using a 25(OH)-vitamin D_3_ direct Enzyme Linked Immunosorbent Assay (ELISA) kit(Immunodiagnostik AG, Stubenwald-Allee, Germany). The method utilizes a competitive ELISA technique with a selected monoclonal antibody recognizing vitamin D. It measures the serum 25(OH)-vitamin D_3_ concentrations in the range of 12–240 nmol/L. The interpretation of the measurement was based on the information from American Society for Bone and Mineral Research (ASBMR) 2006 [34]. A 25(OH)-vitamin D_3_ level ≤25 nmol/L defines severe deficiency whereas < 50 nmol/L defines vitamin D deficiency. The 25(OH)-vitamin D_3_ level between 50 and 75 nmol/L shows insufficiency whereas individuals with > 75 nmol/L of vitamin D have adequate levels.

### Statistical analysis

Data were entered into Epi-info version 3.5.3 and analyzed using Statistical Package for Social Sciences (SPSS) version 20. Data were reported as frequency and percentage for categorical variables and mean and standard deviation (SD) for the normally distributed continuous variables. Categorical variables were analyzed using chi-square test. One-way analysis of variance (ANOVA) with Bonferroni post hoc test was used to determine whether there are any significant differences in the mean serum levels between TB patients, community controls and household contacts. Previous studies reported that only severe vitamin D deficiency (a 25(OH)-vitamin D_3_ level ≤25 nmol/L) not vitamin D deficiency (A 25(OH)-vitamin D_3_ level < 50 nmol/L) was associated with a significantly increased risk of active TB. Therefore, in this study we dichotomized our study participants in to two groups (Individuals with severe vitamin D deficiency and without severe vitamin D deficiency). Binary logistic regression model was fitted to identify factors associated with severe vitamin D deficiency. Variables with a *p*-values of <0.2 in the bivariate analysis were entered to a multivariable binary logistic regression analysis to identify the independent determinants of severe vitamin D deficiency. Both the Crude Odds Ratio (COR) and the Adjusted Odds Ratio (AOR) with a corresponding 95% confidence interval (CI) were calculated to investigate the strength of the association. Statistical significance was considered at 95% level of confidence and *P* value less than 0.05.

## Results

### Socio-demographic and clinical characteristics of the study participants

A total of 126 newly diagnosed tuberculosis patients (78 males and 48 females), 57 household contacts (23 males and 34 females) and 70 community controls (53 males and 17 females) were included in the study. The mean (SD) age of TB patients, household contacts and community controls was 29.8 ± 11.9 years, 24.3 ± 14.7 years and 27.3 ± 7.6 years respectively. A large proportion (72/126 (57.2%)) of tuberculosis patients were from urban areas. The mean BMI was found significantly lower in TB patients (17.4 ± 2.25 kg/m^2^) than in community controls (21.4 ± 2.84 kg/m^2^) (*p* < 0.001) and in household contacts (18.9 ± 3.77 kg/m^2^) (*p* = 0.001). (Table 1).

**Table 1:** Socio-demographic and clinical characteristics of the study participants

Variables	TB patients (*n* = 126) n (%)	Community controls (*n* = 70) n (%)	P*	Household contacts (*n* = 57) n (%)	P**
Gender					
Male	78 (62)	53(76)	0.049	23(40.4)	0.006
Female	48 (38)	17(24)		34(59.6)	
Age (years) mean(SD)	29.8 ± 11.9	27.3 ± 7.6	0.173	24.3 ± 14.7	0.008
BMI,Kg/m^2^ Mean(SD)	17.4 ± 2.25	21.4 ± 2.84	<0.001	18.9 ± 3.77	0.001
Level of education					
Illiterate	38(30)	11(15.7)	0.023	NA	
Read and write	67(53.3)	38(54.3)			
Higher education	21(16.7)	21(30)			
Occupation					
Employed	21(16.7)	28(40)	<0.001		
Unemployed	22(17.5)	10(14.3)		NA	
Daily laborer	28(22.2)	14(20)			
Farmer	36(28.5)	13(18.6)			
Others	19(15.1)	5(7.1)			
Residence					
Urban	72(57.2)	42(60)	0.697	NA	
Rural	54(42.8)	28(40)			
Alcohol use					
Yes	53(42.5)	24(35)	0.285	NA	
No	73(57.5)	46(65)			
Smoking					
Yes	22(17.5)	10(14)	0.564	NA	
No	104(82.5)	60(86)			

P* P for difference among TB patients and community controls; P** P for difference among TB patients and house hold contacts, *NA* not applicable

### Comparison of serum Vitamin D levels between newly diagnosed TB patients, household contacts and community controls

There was a statistically significant difference in the mean serum level of vitamin D between groups as determined by one-way ANOVA (F(df) =6.03(2), *p* = 0.003). A Bonferroni post hoc test revealed that the mean vitamin D level was significantly lower in newly diagnosed TB patients (30.1 ± 19.3 nmol/L) compared to community controls (38.5 ± 20.9 noml/L, *p* =0.005) and house hold contacts (37.7 ± 12.8 nmol/L, *p* =0.031). There was no statistically significant difference in mean vitamin D level between the household contacts and community controls (*p* =0.98) (Table 2).

**Table 2:** Comparison of mean vitamin D levels between newly diagnosed TB patients, household contacts and community controls

Variables	TB patients (*n* = 126)	Community controls (*n* = 70)	P*	Household contacts (*n* = 57)	P**
Vitamin D(nmol/L) Mean ± SD	30.1 ± 19.3	38.5 ± 20.9	0.005	37.7 ± 12.8	0.031

P* P for difference of serum vitamin D level between TB patients and community controls; P** P for difference of serum vitamin D level between TB patients and house hold contacts

### Prevalence of vitamin D deficiency among the study participants

Vitamin D insufficiency (25(OH)-vitamin D_3_ level of 50 to 75 nmol/L) was observed in 11.9% of TB patients, 24.3% of community controls, and 12.7% of household contacts. Using the criteria level of serum 25(OH)-vitamin D_3_ < 50 nmol/L, the prevalence of vitamin D deficiency was 83.3% in patients with TB, and this prevalence was significantly higher than the prevalence in community controls (67.1%, *P* = 0.009). The prevalence of vitamin D deficiency was also found higher in household contacts (80.7%). Severe vitamin D deficiency was observed in 53%(67/126), 30% (21/70), 19.3%(11/57) of TB patients, community controls and household contacts respectively. The observed severe vitamin D deficiency was significantly higher in TB patients than in community controls (*p* = 0.002). (Table 3).

**Table 3:** Proportion of vitamin D deficiency levels among TB patients, community controls and household contacts

Category	TB cases n (%)	Community controls n (%)	P* value	Household contacts n (%)	P** value
Vitamin D deficiency	105(83.3)	47(67.1)	0.009	46(80.7)	0.664
Vitamin D insufficiency	15(11.9)	17(24.3)	0.64	7(12.7)	0.84
Severe vitamin D deficiency	67(53.2)	21(30)	0.002	11(19.3)	<0.001
Normal vitamin D level	6(4.8)	6(8.6)	0.28	1(1.7)	00.32

Chi-square test was used to test for differences in proportions. P* P for difference among TB patients and community controls, P** P for difference among TB patients and house hold contacts*, VDD* Vitamin D deficiency ((25(OH)-vitamin D_3_ ≤ 50 nmol/L))*, VDI* Vitamin D insufficiency ((25(OH)-vitamin D_3_ 51–75 nmol/L)), *SVD* Severe vitamin D deficiency(25(OH)-vitamin D_3_ < 25 nmol/L)

### Risk factors associated with severe vitamin D deficiency among tuberculosis patients and community controls

Logistic regression analysis was performed to identify factors associated with severe vitamin D deficiency. In the crude logistic regression analysis, three factors were associated with severe vitamin D deficiency: low BMI (COR = 2.7; 95% CI = 1.48–4.73;*P* = 0.01), Residence (COR = 0.19 ; 95% CI = 0.05–0.77;*P* = 0.02) and being positive for TB(COR = 2.5 ; 95% CI = 1.36–4.52;*P* = 0.01). In the multivariable binary logistic regression model, low BMI with (*P* =0.001; AOR = 2.13;95% CI = 1.02–3.28) and being positive for TB (*P* = 0.002; AOR = 1.93 ; 95% CI = 1.06–2.86) were independently associated with severe vitamin D deficiency (Table 4).

**Table 4:** Factors associated with severe vitamin D deficiency (vitamin D level <25 nmol/L) among TB patients and community controls, Northwest Ethiopia (*n* =196)

Characteristics	Severe vitamin D deficiency			
	Yes n(%)	No n(%)	COR^a^ (95% CI)	AOR^b^ (95% CI)
Sex				
Male	59(45)	72(55)	1.02(0.56–1.85)	
Female	29(44.6)	36(55.4)	1	
Age				
<18	13(59.1)	9(40.9)	1	
18–30	45(38.8)	71(61.2)	2.27(0.90–5.76)	
31–50	24(50)	24(50)	1.44(0.52–4.01)	
>50	6(60)	4(40)	09.6(0.214.42)	
BMI				
<18.5	56(55.6)	43(43.4)	2.7(1.48–4.73)*	2.13(1.02–3.28)*
>18.5	32(33)	65(67)	1	1
TB status				
TB positive	67(53.2)	59(46.8)	2.5(1.36–4.52)*	1.93(1.06–2.86) *
TB negative	21(30)	49(70)	1	1
Smoking				
Yes	19(59.4)	13(40.6)	0.48(0.08–2.8)	
No	69(42.1)	95(57.9)	1	
Residence				
Urban	69(60.5)	45(39.5)	0.194(0.05–0.8)*	11.3(0.83–153.5)
Rural	19(23.2)	63(76.8)	1	1

*BMI* body mass index

Three variables namely, *BMI* being positive for TB and residence, with a *p-*values of <0.2 in the bivariate analysis were entered to a multivariable binary logistic regression analysis

*P < 0.05

^a^Crude Odds Ratio

^b^Adjusted Odds Ratio

## Discussion

In this study, high prevalence of severe vitamin D deficiency was observed among newly diagnosed TB patients compared to community controls and house hold contacts. In addition, the serum vitamin D level of TB patients was significantly lower than community controls. After controlling for potential confounders using multivariable logistic regression model, low BMI level and being positive for TB were significant predictors of severe vitamin D deficiency.

Similar to the current study, reports from Tanzania [18], Uganda [28] and Malawi [29] show that a low BMI is associated with Vitamin D deficiency in TB patients. This may be explained as patients with low BMI usually have a little adipose tissue so they are unable to store vitamin D, and they have no reserves when there is poor dietary intake of foods that are rich with vitamin D.

The serum vitamin D level of TB patients was significantly lower than community controls. This may be due to the inadequate dietary intake as reflected by the high frequency of low BMI among the newly diagnosed TB patients. More TB patients were unemployed, which indicated a potential lower socioeconomic status of TB patients. It has been suggested that vitamin D deficiency in TB patients might lead to impaired immune control of mycobacteria [9–12]. In line with this, we found that TB was independently associated with severe vitamin D deficiency. Our results are consistent with studies indicating vitamin D deficiency as a risk factor for developing tuberculosis [35]. However, the fact that even household contacts and community controls also have a high level of vitamin D deficiency without association to active TB needs to be further elucidated.

In line with our findings several studies from Tanzania, Vietnam, West Africa, Uganda and Malawi showed higher prevalence of vitamin D deficiency in TB cases than in their corresponding community controls [18, 20, 25, 28, 31]. However, the prevalence of vitamin D deficiency in TB patients in those previous studies was lower than the prevalence in our study. These discrepancies could be explained by the varying study definitions of vitamin D deficiency used, difference in the techniques used for measurement of vitamin D concentrations, the location in terms of latitude of the study sites, seasonal variations, varying dietary habits, frequencies of other co-morbidities and differences in BMI among the respective study participants.

The sunlight is an important source of vitamin D [36, 37], and sun exposure is usually higher in the study area compared to Europe. However, the serum vitamin D level of the study subjects in our study was found to be lower compared to subjects in Greenland [22] and West London [23]. This may be due to differences in skin pigmentation, as melanin efficiently absorbs UVB radiation and dark skin persons require 3 to 4 times longer sun exposure [38]. In addition, it has been reported that skin pigmentation was a significant predictor variable of vitamin D deficiency [39, 40].

Many studies have reported that the prevalence of vitamin D deficiency in tuberculosis patients varies depending on the season [41]. Although our study was carried out during the sunny months, the vitamin D deficiency was still common in TB patients in the current study. The daylight exposure differs little between the summer and a winter months in Ethiopia. Seasonal variation is therefore unlikely to have made a major contribution to the low vitamin D levels.

Our study has some limitations and one of them is common to most other similar studies in that the direct causal relationship between low vitamin D levels and the risk of developing TB needs further investigation. Thus, it is not possible to draw any conclusions about the risk of developing TB in community controls or house-hold contacts based on our data. A follow up study with higher numbers of the community controls and house-hold contacts would have resolved some of these issues. It would also have been of value to include seasonal variation and more clinical and sociodemographic variables, in particular for the house-hold contacts. The major strength of this study is including household contacts for comparison of vitamin D levels to TB patients and that the study was performed in a high endemic area (Ethiopia) where there is very limited knowledge about the association between Vitamin D deficiency and TB. We found a high prevalence of vitamin D deficiency in household contacts of TB patients which may be one factor contributing to the risk of developing TB in such highly exposed individuals.

## Conclusions

High prevalence of vitamin D deficiency was found among newly diagnosed TB patients and house hold contacts. Low body mass index and presence of tuberculosis were associated with severe vitamin D deficiency in newly diagnosed pulmonary tuberculosis patients. Considering the high prevalence of vitamin D deficiency, the present study implicates that further follow-up studies are warranted to determine whether vitamin D supplementation can have a role in the prevention and treatment of tuberculosis in Ethiopia.

## Abbreviations

ANOVA: Analysis of variance
ASBMR: American Society for Bone and Mineral Research
BMI: Body mass index
CC: Community controls
CD4: Cluster of differentiation 4
CD8: Cluster of differentiation 8
DOTS: Directly observed treatment short-course program
ELISA: Enzyme-linked immunosorbent assay
GHC: Gondar Health Center
GUH: University of Gondar Hospital
HHC: Household contacts
HIV: Human immunodeficiency virus
MDR TB: Multiple drug resistant tuberculosis
*Mtb*: *Mycobacterium tuberculosis*
TB: Tuberculosis
VCT: Voluntary counseling and testing
VDR: Vitamin D receptor
WHO: World health organization
